# VITT Pathophysiology: An Update

**DOI:** 10.3390/vaccines13060650

**Published:** 2025-06-17

**Authors:** Eleonora Petito, Paolo Gresele

**Affiliations:** Section of Internal and Cardiovascular Medicine, Department of Medicine and Surgery, University of Perugia, 06132 Perugia, Italy; petitoeleonora@gmail.com

**Keywords:** Anti-PF4 antibodies, HLA, SARS-CoV-2, thrombosis, thrombocytopenia, VITT

## Abstract

Vaccine-induced thrombotic thrombocytopenia (VITT) is a rare thrombotic disorder first identified in 2021 as a catastrophic syndrome associated with anti-SARS-CoV-2 adenoviral vector (AdV)-vaccine administration. It is characterized by the presence of oligo- or monoclonal anti-PF4 antibodies able to induce in vitro platelet activation in the presence of PF4. In addition to this immune-based pathomechanism, random splicing events of the Adv-vector DNA encoding for SARS-CoV-2 spike protein resulting in the secretion of soluble spike variants have been postulated as a possible pathophysiological mechanism. More recently, some novel clinical-pathological anti-PF4-associated entities also characterized by thrombosis, thrombocytopenia, and VITT-like antibodies but independent from heparin or AdV-vaccine administration have been identified. To date, these VITT-like disorders have been reported following the administration of vaccines different from anti-SARS-CoV-2 AdV-vaccines, like human papillomavirus (HPV) and mRNA-based COVID-19 vaccines, following a bacterial or viral respiratory infection, and in patients with a monoclonal gammopathy of undetermined significance. The purpose of this review is to provide an update on the knowledge on VITT pathogenesis, focusing on recent findings on anti-PF4 antibodies, on a possible genetic predisposition to VITT, on VITT-antibody intracellular activated pathways, on lipid metabolism alterations, and on new VITT-like disorders.

## 1. Introduction

Vaccine-induced thrombotic thrombocytopenia (VITT) is a thrombotic disorder first identified in 2021 as a catastrophic syndrome associated with anti-SARS-CoV-2 adenoviral vector (AdV)-vaccine administration characterized by high mortality and the need for urgent treatment [1,2,3]. The incidence of VITT is estimated to be 3 to 15 cases per million initial vaccinations [4]. While most VITT cases occurred after the first vaccine dose, there have been reports following the second administration. These secondary cases appear to be less frequent, with a more rapid onset and a milder progression [5]. 

No predisposing risk factors have been identified so far, and the higher incidence in young women initially described [1,6,7] has probably been caused by the decision to vaccinate first healthcare workers who are in many countries more frequently females. The syndrome is mediated by antibodies (Abs) of the immunoglobulin G (IgG) class, the majority belonging to the IgG1 subclass, that target the highly positively charged chemokine platelet factor 4 (PF4) forming immune complexes that trigger a multicellular thromboinflammatory storm similar, although not identical, to heparin-induced thrombocytopenia (HIT) [8]. VITT antibodies trigger FcγRIIA-mediated activation of platelets and neutrophils, which play the main pathogenic role in VITT and accumulate on the surface of endothelial cells by binding to PF4 linked to endothelial glycosaminoglycans, thereby activating them and amplifying the thromboinflammatory response [9,10]. Given the rare occurrence of VITT, a possible genetic predisposition has been suspected [11]. More recently, some novel clinical-pathological thrombotic thrombocytopenic syndromes (TTS) associated with anti-PF4 antibodies but independent from heparin or AdV-vaccine administration, called VITT-like disorders, have been identified. Moreover, patients who developed VITT following anti-SARS-CoV-2 mRNA vaccine administration but with no detectable anti-PF4 antibodies have also been characterized, suggesting that other IgG complexes can also induce FcγRIIa-dependent platelet activation [12,13,14,15].

The purpose of this review is to provide an update on VITT pathogenesis, with a special focus on recent findings and new VITT-like disorders.

## 2. Terminology

*Thrombosis with thrombocytopenia syndromes (TTS)* has been used as a general descriptive name indicating a rare, acute, and serious medical condition characterized by thrombosis, often in unusual sites and/or multiple, associated with a low platelet count. It is often immune-mediated due to an abnormal immune response to a trigger, such as vaccination or infections, that activates platelets. It includes different entities, such as VITT and anti-PF4-negative TTS, in subjects who had received mRNA-based COVID-19 vaccines [15].

*PF4-associated immune thrombocytopenia and thrombosis (PITT)* has been proposed as a collective name for heparin-induced thrombocytopenia (HIT), VITT, and VITT-like disorders [16,17].

*Vaccine-induced thrombotic thrombocytopenia (VITT)*: thrombotic disorder first identified in 2021 as a catastrophic syndrome associated with anti-SARS-CoV-2 adenoviral vector (AdV)-vaccine administration characterized by the presence of anti-PF4 antibodies [18].

*VITT-like disorders*: novel thrombotic thrombocytopenic entities associated with anti-PF4 antibodies but independent from heparin or AdV-vaccine administration. To date, they have been reported following the administration of vaccines different from AdV-vaccine anti-SARS-CoV-2, like human papillomavirus (HPV) and mRNA-based COVID-19 vaccines, following bacterial or viral respiratory infections, and in patients with monoclonal gammopathy of undetermined significance [18].

## 3. The Pathogenesis of VITT

Key features of VITT are unusual site venous thrombosis, predominantly cerebral venous sinus (CVST) and splanchnic vein thrombosis (SVT), frequently accompanied by multiple venous and/or arterial clots, severe thrombocytopenia, a striking rise in D-dimer levels with signs of disseminated intravascular coagulation (DIC) associated with the presence of anti-platelet factor 4 (PF4)/heparin complex antibodies in the absence of prior exposure to heparin, occurring 5–30 days after anti-SARS-CoV-2 vaccination [3,12,19]. Recently, case definitions for VITT have been proposed and validated [18,20]. Anti-PF4 antibodies have been defined as the pathognomonic laboratory marker of the disease [1,3,12,19]. These antibodies target the positively charged platelet-derived chemokine PF4, similar to HIT antibodies; however, the epitope recognized on PF4 and antibody profile differ. Alanine-scanning mutagenesis pinpointed the binding site of antibodies from ChAdOx1-vaccinated patients to a compact, eight-aminoacid epitope within the PF4’s heparin-binding site. This contrasts with HIT antibodies, which target a larger, thirteen-aminoacid region across two distinct epitopes. Moreover, differently from HIT antibodies that require heparin to induce platelet activation in vitro (heparin-dependent antibodies), VITT antibodies bind strongly to PF4 in the absence of heparin (heparin-independent antibodies) [21], and a precise stoichiometric ratio between PF4 and anti-PF4 antibodies is required for platelet activation in fact, contrary to HIT antibodies, diluting VITT serum may increase the probability of a positive platelet activation assay [22]. Furthermore, biolayer interferometry demonstrated a significantly higher binding affinity for PF4 of VITT antibodies compared to HIT antibodies [21]. Subsequent studies confirmed the restricted epitope specificity and high binding affinity also for VITT antibodies from Ad26.COV2.S-vaccinated patients [23]. Unlike HIT antibodies, which are polyclonal, VITT antibodies analyzed by liquid chromatography-electrospray ionization quadrupole time-of-flight mass spectrometry (LC-ESI-QTOF MS) exhibit monoclonal or oligoclonal light chains [24]. In addition, anti-PF4 antibodies belonged to the immunoglobulin G1 (IgG1) and IgG2 subclass, and all contained λ light chains [24,25]. Mass spectrometry-based proteomics identified highly stereotypic antibodies consisting of an identical light chain encoded by the IGLV3-21*02 allele paired with a shared heavy chain third complementarity–determining region (HCDR3) [26]. Moreover, mass spectrometry of a monoclonal anti-PF4 antibody (mAb) extracted from a VITT patient’s blood revealed that it has a fully mature biantennary N-glycan within its V_H_ domain. This novel N-glycosylation site has been reported recently to be shared by several autoimmune disorders, suggesting that this feature may act as a molecular trigger that activates noncanonical clone selection pathways and allows to evade elimination by the “self/not-self” control machinery, leading to the onset of autoimmunity [25]. From all the above studies, it thus emerges that VITT autoantibodies are unique and seem to be restricted to specific subsets of individuals, e.g., those with a specific IgG light chain hypervariable region. 

Based on in vitro and animal studies, a theory on the pathogenic mechanisms of VITT has been formulated, envisaging the entry of negatively charged vaccine components into the bloodstream, either by an unintentional intravenous injection or facilitated by the increase in endothelial permeability provoked by the EDTA excipient of ChAdOx1 nCoV, which then interact with positively charged PF4 forming complexes which induce a conformational change in PF4 triggering the activation of B cells, that then start to produce autoantibodies against PF4 (Table 1). Immune complexes between PF4 and anti-PF4 antibodies bind to platelet and neutrophil FcγRIIA, generating procoagulant platelets and stimulating the release of neutrophil extracellular trap (NET) thus eliciting thrombosis [3,8,11]. Surface plasmon resonance has shown the high binding affinity of the purified hexon protein of the Adv capsid, present in ChAdOx1, for PF4 as a result of electrostatic interactions [10,27]. However, which are the specific vaccine constituent(s) triggering the anti-PF4 response and why immune tolerance is broken, resulting in anti-PF4 antibody production, needs to be further investigated. Indeed, no direct binding between PF4 and another largely used anti-SARS-CoV-2 AdV-based vaccine, Ad26.COV.2.S was shown using several biophysical techniques, suggesting that it is very unlikely that this direct interaction is sufficient for the onset of VITT [28].

An alternative pathophysiological hypothesis to explain why only DNA-based, and not RNA-based, vaccines trigger VITT has been postulated. SARS-CoV-2 is a single-stranded RNA virus that is translated and replicated in the cytoplasm of infected cells without being integrated into nuclear DNA (Table 1). When the viral RNA sequence of the spike protein is delivered via a DNA adenoviral vector instead, the adenoviral DNA integrates into the host cell nucleus and is subsequently transcribed into the immunizing protein. However, while the single-stranded RNA of SARS-CoV-2 does not undergo splicing, eukaryotic genes undergo random splice events required to remove intronic sequences. As a result, random splicing of the integrated DNA encoding the spike protein may lead to the generation of abnormal mRNAs encoding for spike protein variants, some of which may lack the transmembrane anchor, resulting in the release of soluble spike copies circulating in the blood. This hypothesis could explain the higher frequency of VITT after vaccination with ChAdOx1 nCoV-19 than after Ad26.COV2.S, given that the latter carries fewer splice donor sequences compared to ChAdOx1 nCoV-19 [29]. The binding of circulating soluble spike to endothelial ACE2 would then become a target of anti-spike antibodies generated by vaccination, triggering a vascular inflammatory response leading to thrombosis. The absence of valves in the cerebral sinus veins, allowing bi-directional, posture-dependent blood flow, would prolong the residence time of soluble spike in that vascular district, thereby increasing the likelihood of binding to endothelial ACE2, thus triggering thrombosis. Confirmatory findings are that random splicing and generation of soluble spikes have been demonstrated in human cell lines such as HEK293T, HeLa, HepG2, and C2C12 transduced with ChAdOx1 nCoV-19 or Ad26.COV2.S. Moreover, soluble spike protein was detected in sera of VITT patients positive for anti-PF4 antibodies [30]. The circulating SARS-CoV-2 soluble spike protein contributed to ChAdOx1 nCoV-19 vaccine-induced platelet activation, given that the addition of an antibody against SARS-CoV-2 spike decreased VITT serum-induced platelet activation [30]. Limitations to this hypothesis are that the aberrant splice transcripts were found to be rare and were identified only in cell lines not derived from muscle, while these vaccines are administered intramuscularly [29,31]. Moreover, the soluble spike was also detected in sera of ChAdOx1 nCoV-19 vaccinated subjects not developing VITT, and their sera were unable to induce in vitro platelet activation.

**Table 1:** Pathogenic mechanisms proposed for VITT caused by anti-SARS-CoV-2 vaccines.

Vaccine	Anti-PF4 Abs	Pathogenetic Mechanism	Involved Receptors	Target Cells	References
AdV-basedanti-SARS-CoV-2 vaccine	Positive	Anti-PF4 antibodies	FcγRIIA	PlateletsNeutrophils	[8]
AdV-basedanti-SARS-CoV-2 vaccine	Positive	Generation of soluble SARS-CoV-2 spike protein caused by unwanted splicing events	ACE-2	Endothelial cells	[29,30]
mRNA-basedanti-SARS-CoV-2 vaccine	Negative	Spike/anti-spike IgG complexes	FcγRIIA	Platelets	[14]
mRNA-basedanti-SARS-CoV-2 vaccine	Negative	Histone/anti-histone IgG complexes	FcγRIIA	Platelets	[15]

Thus, a multiple-hit model merging the two pathogenic theories has been proposed. The first hit would involve the interaction between soluble spike and ACE2 receptors on endothelial cells, leading to platelet recruitment, activation, and the release of PF4. Released PF4 would then interact with vaccine polyanions, generating new antigens that trigger the production of anti-PF4/polyanion autoantibodies and the formation of immune complexes. The second hit would then occur when these immune complexes bind to FcγRIIA receptors on platelets and neutrophils, resulting in their activation and thrombus formation [30].

## 4. TTS Negative for Anti-PF4 Antibodies

Since the first reported VITT series, a few patients negative for anti-PF4 antibodies were described. Among 105 VITT patients reported in the literature as of July 2021, 5 were negative for anti-PF4 antibodies [32]. As of 31 October 2022, 56 cases of VITT following ChAdOx1 nCoV-19 vaccine administration were identified by the Canadian Adverse Events Following Immunization Surveillance System (CAEFISS), 6 of which were negative for anti-PF4 antibodies evaluated by PF4-enhanced serotonin release assay [13]. 19 out of 40 VITT patients enrolled in Australia between April and September 2021 were negative for anti-PF4 antibodies, evaluated by PF4/polyanion ELISA, but still, their sera were able to induce platelet deposition on endothelial cells [9]. These results can, in part, be explained by the different sensitivity of anti-PF4 detection assays, in particular of various ELISA platforms, underlining the importance of assessing more than one ELISA and a functional assay showing the platelet-activating activity of VITT antibodies [33,34,35]. However, it cannot be excluded that other immunopathogenic mechanisms, beyond anti-PF4 antibodies, may cause VITT. Recently, platelet-activating spike/anti-spike and histone/anti-histone IgG complexes have been associated with some VITT cases that occurred after mRNA COVID-19 vaccination whose sera tested negative for anti-PF4 antibodies but were still able to induce FcγRIIA-mediated platelet activation (Table 1) [14,15]. The authors suggested a double hit model: anti-SARS-CoV-2 mRNA-based vaccines (or other proinflammatory processes) trigger activation of macrophages, monocytes, and granulocytes inducing the release of histones with the consequent formation of anti-histone autoAbs (first hit). A second trigger (vaccination or other processes) would then induce new histone release, boosting antibody synthesis and leading to the rapid formation of immune complexes between circulating histones and anti-histone antibodies (second hit). These complexes would activate platelets, resulting in VITT [15]. Intriguingly, VITT sera negative for both anti-PF4 and anti-histone antibodies but still able to induce the generation of procoagulant platelets via FcγRIIa activation have also been reported, suggesting that additional platelet activating immune complexes may also exist [15].

## 5. Recent Acquisitions on VITT Pathogenesis

In this section, we aim to summarize the more recent knowledge on VITT pathogenesis.

### 5.1. Anti-PF4 Antibodies

Given the limited availability of samples from VITT patients, recombinant antibodies (rAbs) based on anti-PF4 proteomes from sera of VITT patients were generated, using a novel reverse-engineering approach that bypasses conventional B-cell sorting and cloning methods, and characterized [36,37]. The full-length anti-PF4 IgG proteins were affinity-purified from sera of patients with VITT associated with anti-SARS-CoV-2 AdV-vaccine administration using PF4 bound to a solid phase [26], then amino acid sequenced by mass spectrometry and finally expressed in CHO cells, thus creating a library of stereotypic recombinant anti-PF4 Abs. Light chains of all generated rAbs were encoded by the same IGLV3-21*02 allele, and the generated rAbs recapitulated the properties of Abs isolated from VITT patients with similar PF4 specificity, binding characteristics, and functional impact. In fact, they bound epitopes in the heparin-binding site of PF4, formed immune complexes with PF4, and activated platelets in a FcγRIIa-dependent manner [36].

### 5.2. Genetic Predisposition to VITT

The above-mentioned VITT antibody fingerprint [26] may suggest a genetic predisposition to VITT. This has been investigated by a high-throughput whole exome sequencing (WES) approach in six Italian patients with a definite diagnosis of VITT [38]. The authors did not find any significant enrichment of mutant genes common to the enrolled subjects. Thus, they focused their attention on variants in genes with a role in blood coagulation and fibrinolysis, platelet activation and aggregation, integrin-mediated signaling, inflammation, and autoimmune thrombocytopenia. Altogether, in the six patients, 194 rare variants were identified, all in the heterozygous state, but none of them was classified as likely pathogenic or pathogenic [38]. Another study in 16 AdV-COVID vaccine-associated VITT patients, 11 of whom definite and 5 probable, did not identify in VITT patients an accumulation of rare single nucleotide variants compared to a large cohort of 402 healthy controls, thereby excluding an association between rare gene variants and the risk of suffering from VITT [39]. 

The FCGR2A H131R polymorphism, a single nucleotide polymorphism of FcγRIIA regulating the affinity for IgG, was found in a homozygous state in two young male first cousins who both suffered from VITT after Ad26.COV2.S, prompting considerations about a possible familial genetic predisposition to VITT [40]. Moreover, the MTHFR thrombophilic mutation C677T has been reported in 3 patients who suffered VITT after receiving the first dose of ChAdOx1, 2 of whom were homozygous and 1 heterozygous [41,42,43]. However, both the FCGR2A H131R and the C677T variants occur with high frequency in the general population; thus, the possibility that this was a chance association is very high. In fact, according to the gnomAD database, 23% of the population is homozygous for FCGR2A rs1801274 H131R, and according to the 1000 Genomes Project, approximately 25% of the global population are carriers of MTHFR C677T variant [44].

An association between specific Human Leukocyte Antigen (HLA) class II alleles and VITT has been recently reported in a case–control study in sixteen unrelated Caucasian Italian VITT patients [39]. Three HLA class II alleles were detected with significantly higher frequency in VITT patients compared with 198 Italian controls: HLA-DPB1*17:01 (0.125 in VITT vs. 0.002 in controls, *p* = 0.0009), HLA-DQA1*05:01 (0.375 in VITT vs. 0.085 in controls, *p* = 0.00015) and HLA-DRB1*11:04 (0.218 in VITT vs. 0.053 in controls, *p* = 0.03380). In addition, a specific PF4-derived peptide was identified as a strong binder for one of these alleles, HLA-DRB1*11:04, by in silico analysis using the NetMHCIIpan-4.3 software able to calculate the binding affinity of human PF4 peptides for the VITT related HLA class II alleles using neural networks. Interestingly, this peptide contained two amino acids belonging to the specific 8 aminoacid-PF4 epitope recognized by VITT antibodies [39]. 

These findings remind of previous studies reporting an association between some HLA alleles, particularly those of class II, with the risk of developing other thrombotic thrombocytopenic autoimmune disorders, like thrombotic thrombocytopenic purpura (TTP), heparin-induced thrombocytopenia (HIT) and immune thrombocytopenia (ITP) [45,46,47]. It can thus be hypothesized that HLA-restricted antigen presentation may play a role in the formation of anti-PF4 antibodies, predisposing to the development of VITT [46,47]. 

Certainly, this study needs validation in larger case series; however, it supports the hypothesis that the VITT immune response is oligoclonal or potentially monoclonal, suggesting that a single or a limited number of leukocyte clones may be responsible for the production of VITT antibodies [48]. While for the future mass HLA-typing of subjects candidate to Ad vector vaccines is likely unfeasible, this might be of help to prevent thrombotic complications related to the use of adenoviral vector platforms for gene therapies [19].

### 5.3. Cell Activation Pathways

The activating effect of anti-PF4 VITT antibodies on platelets and neutrophils via FcγRIIA is the key trigger of immunothrombosis in VITT [3]. Recently, the cellular molecular pathways activated by FcγRIIA have been better characterized, unraveling potential new therapeutic approaches.

#### 5.3.1. Endothelial Cell Activation

Endothelial activation is an important player contributing to the development of thromboinflammation in VITT. The expression of the vascular cell adhesion molecule 1 (VCAM-1) and the deposition of proinflammatory complement components on the vascular endothelial surface have been documented by immunohistochemistry in the microcirculation of the heart, lung, liver, kidney, and ileum in patients who died from VITT after ChAdOx1 nCoV-19 vaccination [49]. Using a novel endothelialized microfluidic chip, serum or isolated IgGs from VITT patients were found to induce an increase in the endothelial expression of tissue factor (TF), with a consequent enhancement of platelet, neutrophil, and fibrin deposition, a phenomenon magnified by PF4. This increased deposition was blunted by the inhibition of endothelial TF using an inhibitory anti-TF antibody or by the blockade of FcγRIIA on platelets and neutrophils using the monoclonal antibody IV.3 [9]. In addition, treatment of endothelial cells with VITT serum led to a significant increase in the surface expression of endothelial activation markers, such as P-selectin and VCAM-1, as well as in von Willebrand factor (VWF) secretion, all phenomena enhanced in the presence of PF4 [9]. Given that ChAdOx1 is able to transduce endothelial cells in vitro, despite lacking the primary cell entry receptor Coxsackie and Adenovirus Receptor (CAR), perhaps through αvβ3 and αvβ5 integrins, heparan sulfate proteoglycans or CD46 [10], it was supposed that this might have a role in VITT pathogenesis. However, the direct transduction of endothelial cells with ChAdOx1 did not seem to contribute to the pathogenesis of VITT, given that it did not induce endothelial cell activation [10].

#### 5.3.2. Platelet-Neutrophil Aggregate Formation and Inflammasome Activation

Platelet-leukocyte aggregate formation and inflammasome activation are typical features of thromboinflammatory diseases [50]. The central role of neutrophil activation and the consequent release of NETs in VITT pathogenesis emerged since the first description of VITT by the detection of degranulated neutrophils as well as of NET biomarkers in circulating blood and in platelet-rich CVS thrombi, suggesting a causal relationship between prothrombotic NETs and VITT [8,51]. Indeed, IgG isolated from VITT patients directly stimulates neutrophils to release NETs and induce thrombus formation in vitro in a microfluidic flow system composed of microchannels coated with VWF, as well as in vivo in double transgenic mice (FcγRIIa^+^/hPF4^+^) in which VITT was triggered by the intravenous injection of purified VITT IgG [51]. The blockade of FcγRIIa abrogated thrombosis and thrombocytopenia in vivo [51], highlighting the key pathogenic role of the interaction between VITT IgG-PF4 complexes and FcγRIIa. Moreover, plasma from VITT patients induced platelet-neutrophil aggregate formation in vitro in a FcγRIIa-dependent manner, associated with neutrophil NLRP3 inflammasome activation evaluated by caspase-1 activation [52]. Concordant with these findings, plasmatic levels of caspase 1 and IL-1β were found to be increased in VITT patients compared to unvaccinated controls [52]. Thus, the prothrombotic state of VITT patients is supported by NLRP3 inflammasome activation mediated by FcγRIIa-dependent platelet-neutrophil aggregate formation.

#### 5.3.3. Platelet Spleen Tyrosine Kinase (SYK)

VITT antibodies induce a procoagulant platelet phenotype, evaluated as increased expression of P-selectin and phosphatidylserine, that drives the crosstalk between platelets and neutrophils and the activation of the plasmatic coagulation system. The selective inhibition of SYK, a key signaling enzyme downstream of FcγRIIA, abolished VITT IgG-induced procoagulant platelet formation, platelet-neutrophil crosstalk, thrombus formation, and the activation of the plasmatic coagulation system [53]. These data demonstrate the key role of platelet FcγRIIA-associated SYK activation in the induction of immunothrombosis in VITT, suggesting that the specific targeting of platelet SYK may be a promising therapeutic strategy for VITT [53].

#### 5.3.4. Lipid Metabolism

The role of lipids in the pathogenesis of arterial and venous thromboembolism (VTE) is well established, with plasmatic cholesterol and triglyceride levels associated with atherothrombosis and cardiovascular disease and a variety of less abundant soluble plasma lipids affecting thrombin generation [54,55,56,57]. A distinct plasmatic lipidomics signature, which differs both from individuals who received the ChAdOx1 vaccine without developing VITT and from patients with standard unprovoked venous thromboembolism (VTE), has been reported in 17 VITT samples. Plasmatic lipid profile was characterized by elevations in phosphatidylserine and ceramide species, a subclass of sphingolipids, together with reductions in several plasmalogens, natural antioxidants, and acylcarnitine species. Phosphatidylserine acts as a potent prothrombinase cofactor, promoting procoagulant activity and thrombin generation when exposed to platelet membranes, and ceramides contribute to the generation of reactive oxygen species and endothelial dysfunction. Indeed, acylcarnitine species have an anticoagulant role. These results are consistent with the prothrombotic nature of VITT, and the existence of this unique VITT lipid signature, which differs from that of standard unprovoked VTE, may offer novel insights into the pathophysiology of VITT [58].

## 6. New VITT-like Disorders

**Figure 1:**
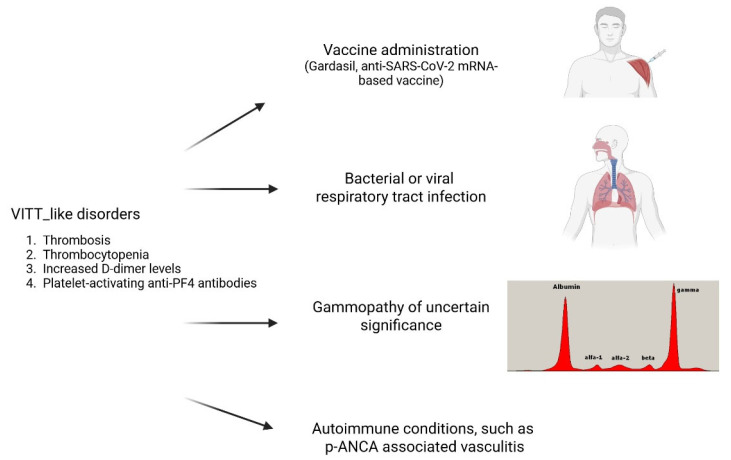
Reported causes of new-VITT-like disorders.

The dogma characterizing VITT exclusively as an AdV-vaccine-associated syndrome is now being replaced by a more complex scenario. There is growing evidence that anti-PF4 antibodies can lead to severe thrombotic disorders not only in the absence of heparin treatment but also in the administration of anti-SARS-CoV-2 AdV vaccines. Recent case reports and retrospective analyses have led to the proposal of a new disease category called “new VITT-like disorders”, sharing the clinical and laboratory pathognomonic features of VITT, including thrombocytopenia, catastrophic thrombosis, strikingly increased D-dimer levels, and positivity for platelet-activating anti-PF4 antibodies, but associated with different triggers. The causes so far reported of this new entity are summarized in Figure 1. They include the administration of vaccines different from the anti-SARS-CoV-2 AdV-vaccines, like Gardasil 9 for human papillomavirus (HPV), a recombinant vaccine using purified virus-like particles constructed from the L1 protein of specific HPV types, and mRNA-based COVID-19 vaccines [15,59,60,61,62]. Examples are a 25-year-old woman with thrombocytopenia, right internal iliac vein thrombosis and pulmonary embolism, elevated D-dimer, and high levels of platelet-activating antibodies to platelet factor 4-polyanion complexes developing 10 days after the administration of Gardasil 9 [59,60]. Moreover, several cases of VITT following anti-SARS-CoV-2 mRNA-based vaccine administration positive for anti-PF4 antibodies have also been reported [61,62,63]. In addition, cases of VITT-like disorders have also been associated with a recent infection, either bacterial or viral [11,64,65,66,67,68]. Examples are a 35-year-old woman with CVST, thrombocytopenia, high D-dimer levels, and anti-PF4 antibodies with a strongly positive PF4-dependent but negative heparin-dependent platelet activation test who died from a fatal secondary intracerebral bleeding. She had suffered two weeks before an upper respiratory tract infection [11]. Nine patients were identified as VITT-like due to unusual thromboses (specifically CVST, SVT, and arterial events), thrombocytopenia, high D-dimer levels, and the presence of anti-PF4 IgG antibodies. Moreover, they exhibited a strongly positive PF4-induced platelet activation (PIPA) test and a negative heparin-induced platelet activation (HIPA) test. None of these patients had heparin exposure or vaccination in the three months prior to the thrombotic event, however the 55.6% of whom experienced an infection before their thrombotic episode [64]. Six cases, five pediatric and one adult, of VITT-like disorders developing around one week after adenoviral infection were recently published [65,66,67,68], one pediatric with a recent parainfluenza virus infection [68], one with group A streptococcal infection [67] and a third with an upper respiratory infection with no identified infective agent [68]. The antibody clonotype of 4 patients with post-adenoviral infection VITT-like disorders analyzed by mass spectrometric sequencing [26] was highly similar to that previously observed in patients with postvaccination VITT [69]. This remarkable similarity in autoantibody fingerprints suggests that VITT and the anti-PF4 disorder linked to adenoviral infections may represent a unique category of adverse immune responses related to adenoviral structures. Recently the case report of a 37 years old woman, who, despite no history of vaccination or heparin exposure, was hospitalized with portal vein thrombosis and thrombocytopenia that worsened upon heparin exposure, culminating in her death, was reported [16]. The patient exhibited high titer anti-PF4 antibodies and an acute cytomegalovirus infection. Interestingly, anti-PF4 antibodies showed both VITT-like and HIT-like behavior depending on the functional assay used [16]. Epitope mapping and proteomic analysis by mass-spectrometry revealed that these anti-PF4 antibodies recognized both known VITT- and HIT-antibody binding sites on PF4, as well as a novel epitope on PF4, and displayed a distinct clonotype compared to VITT antibodies [16,26,69].

Recently, a thrombotic thrombocytopenic disorder was observed in 9 patients with monoclonal gammopathy of undetermined significance (MGUS), associated with platelet-activating monoclonal anti-platelet factor 4 (PF4) antibodies [70,71,72,73,74]. This condition was characterized by recurrent thromboses and thrombocytopenia, positivity for the platelet-activating anti-PF4 antibodies, and was defined as monoclonal gammopathy of thrombotic significance (MGTS) [70,71,72,73,74]. The serum monoclonal paraprotein of MGUS, the M-protein, and monoclonal anti-PF4/polyanion antibody light and heavy chains had identical mass spectrometry profiles, suggesting that these paraproteins were anti-PF4 antibodies [71]. Interestingly, the antibody clonotype profiles and epitope mapping on PF4 by alanine scanning mutagenesis were different from those observed with the post-AdV-vaccination VITT or post-adenoviral infection, suggesting a distinct immunopathogenesis [73]. Moreover, a typical VITT-like syndrome with CVST, thrombocytopenia, and hyperfibrinolysis was reported in a female neonate due to transplacental transfer of maternal platelet-activating prothrombotic anti-PF4 IgG that had caused pulmonary embolism in a previous pregnancy 18 months before [75]. Finally, an 86-year-old woman with anti-PF4 mediated immune thrombosis, occurring in the absence of prior heparin exposure, associated with crescentic glomerulonephritis secondary to perinuclear anti-neutrophil cytoplasmic antibody (p-ANCA)-associated vasculitis, was reported [76]. Serum NETosis markers were significantly elevated at the time of the diagnosis of p-ANCA-associated vasculitis, with a further increase during the complication of anti-PF4 mediated immune-thrombosis, highlighting the need for targeted strategies to address dysregulated NETosis in this pathological context. The resolution of both anti-PF4-mediated immune-thrombosis and NETosis was achieved by intravenous immunoglobulin (IVIG) treatment [76].

In addition to these recent case reports, a retrospective analysis conducted on 188 consecutive patients with thrombocytopenia and/or thrombosis studied before the COVID-19 pandemic and with a laboratory pattern suggestive of VITT, i.e., the presence of anti-PF4/heparin IgG, showed that 13 tested positive by PIPA, highlighting the existence of VITT-like antibodies even before the COVID-19 pandemic and the vaccination campaign [64].

Given the widening spectrum of anti-PF4-mediated TTS, a new collective name for HIT, VITT, and VITT-like disorders has been proposed: PF4-associated immune thrombocytopenia and thrombosis (PITT) [16,17]. PITT should be considered in all patients presenting with thrombocytopenia, thrombosis, and high D-dimer level (>4000 ng/mL), even without preceding vaccination or heparin. Initial treatment with non-heparin anticoagulation has been proposed if PITT is suspected before the subtype is identified [16,17]. A summary of the main diagnostic aspects of VITT and VITT-like syndromes and of treatment options reported for VITT-like syndromes are reported in Table 2 and Table 3. 

## 7. Conclusions and Future Directions

Recent acquisitions reveal that VITT is more heterogeneous and has a broader impact than previously thought; thus, although AdV-vaccines anti-SARS-CoV-2 have been abandoned, we should still pay attention to VITT. The expanding spectrum of VITT-like disorders prompts further studies to better understand the mechanisms underlying thrombotic thrombocytopenic anti-PF4-triggered immune disorders. Several questions regarding VITT and VITT-like disorders remain to be addressed. These include the validation in larger case series of the reported association between specific HLA alleles and the risk of developing these autoimmune disorders, the search for possible specific blockers of the HLA presenting PF4-derived peptides potentially preventing inappropriate immune responses [77], the clonality of pathogenic anti-PF4 antibodies, the intracellular signaling pathways involved in the detrimental effects of anti-PF4 antibodies, and the effects of persisting anti-PF4 antibodies on future exposure to heparin, adenovirus-based vaccines, or viral infections. Special attention should be paid to the potential existence of other antigens, aside from PF4 and hexons, that may trigger the pathogenic autoimmune response through further proteomic studies using mass spectrometry. The existence of VITT sera negative for both anti-PF4 and anti-histone antibodies but still able to induce the generation of procoagulant platelets via FcγRIIa activation suggests that additional platelet activating immune complexes may also exist, and these should be identified. Moreover, a special focus should be placed on investigating anti-PF4-mediated immunothrombosis without prior heparin exposure in patients with autoimmune conditions. All these efforts could significantly contribute to identifying new therapeutic targets and improving the management of patients with unexplained thrombosis and thrombocytopenia, including those who test negative for anti-PF4 antibodies, and may help to prevent thrombotic complications related to the use of adenoviral vector platforms for gene therapies through HLA-typing of candidate subjects.

## Figures and Tables

**Table 2:** Diagnostic aspects of VITT, VITT-like syndromes, and TTS.

	VITT	VITT-like Syndromes	TTS
Thrombocytopenia	Yes	Yes	Yes
Thrombosis at unusual sites	Yes	Yes	Yes
Proximate heparin administration	No	No	No
Proximate anti-SARS-CoV-2 vaccine administration	Yes	No	Yes/No
D-dimer level > 0.5 mg/L FEU	Yes	Yes	Yes
Anti-PF4 antibodies (ELISA)	Yes	Yes	Yes/No *
Platelet-activation assay (PIFPA, PIPA, SRA)	Yes	Yes	Yes/No *

* = references [14,15]. ELISA: enzyme-linked immunosorbent assay; FEU: fibrinogen equivalent units; PIFPA: PF4- induced flow cytometry-based platelet activation (PIFPA) assays; PIPA: PF4-induced platelet activation (PIPA); SRA: serotonin release assay.

**Table 3:** Treatment options reported for VITT-like syndromes.

Treatment	References
Methylprednisolone (1 mg/kg/day)	[16,63,67]
IVIg (1 gr/kg/day)	[59,61,62,63,65]
Platelet transfusions	[65,66,67]
Prednisone oral (1 mg/kg/day)	[59,66]
Plasma exchange	[64,66]
Non-heparin anticoagulant (Fondaparinux, Bivalirudin, or Argatroban)	[59,62,64,66]
Direct Oral Anticoagulants (DOAC)	[59,63,64]
Unfractionated heparin (UFH)	[61,64,65]
